# A Balanced Diet Is Necessary for Proper Entrainment Signals of the Mouse Liver Clock

**DOI:** 10.1371/journal.pone.0006909

**Published:** 2009-09-07

**Authors:** Akiko Hirao, Yu Tahara, Ichiro Kimura, Shigenobu Shibata

**Affiliations:** 1 Laboratory of Physiology and Pharmacology, School of Advanced Science and Engineering, Waseda University, Shinjuku-ku, Tokyo, Japan; 2 Laboratory of Developmental Biology, School of Human Sciences, Waseda University, Saitama, Japan; Vanderbilt University, United States of America

## Abstract

**Background:**

The peripheral circadian clock in mice is entrained not only by light-dark cycles but also by daily restricted feeding schedules. Behavioral and cell culture experiments suggest an increase in glucose level as a factor in such feeding-induced entrainment. For application of feeding-induced entrainment in humans, nutrient content and dietary variations should be considered.

**Principal Finding:**

To elucidate the food composition necessary for dietary entrainment, we examined whether complete or partial substitution of dietary nutrients affected phase shifts in liver clocks of mice. Compared with fasting mice or *ad libitum* fed mice, the liver bioluminescence rhythm advanced by 3–4 h on the middle day in *Per2*::luciferase knock-in mice that were administered a standard mouse diet, i.e. AIN-93M formula [0.6–0.85 g/10 g mouse BW] (composition: 14% casein, 47% cornstarch, 15% gelatinized cornstarch, 10% sugar, 4% soybean oil, and 10% other [fiber, vitamins, minerals, etc.]), for 2 days. When each nutrient was tested alone (100% nutrient), an insignificant weak phase advance was found to be induced by cornstarch and soybean oil, but almost no phase advance was induced by gelatinized cornstarch, high-amylose cornstarch, glucose, sucrose, or casein. A combination of glucose and casein without oil, vitamin, or fiber caused a significant phase advance. When cornstarch in AIN-93M was substituted with glucose, sucrose, fructose, polydextrose, high-amylose cornstarch, or gelatinized cornstarch, the amplitude of phase advance paralleled the increase in blood glucose concentration.

**Conclusions:**

Our results strongly suggest the following: (1) balanced diets containing carbohydrates/sugars and proteins are good for restricted feeding-induced entrainment of the peripheral circadian clock and (2) a balanced diet that increases blood glucose, but not by sugar alone, is suitable for entrainment. These findings may assist in the development of dietary recommendations for on-board meals served to air travelers and shift workers to reduce jet lag-like symptoms.

## Introduction

Various physiological phenomena such as body temperature, food intake, and the sleep-wake cycle are under the control of an endogenous circadian clock [1]. Clock genes such as *Per1, Per2*, *Clock*, *Bmal1*, *Cry1*, and *Cry2* are expressed not only in the central autonomic clock, i.e., the suprachiasmatic nucleus (SCN) of the hypothalamus, but also in other brain regions [2] and various peripheral tissues [3]–[4], including the heart, liver, and kidney. Light-dark (LD) signals through the retina can entrain the SCN circadian clock as well as the peripheral circadian clock through output signals from the SCN. In normal animals, light-driven diurnal behavior can be easily overcome by alterations in energy supply under conditions such as restricted feeding schedules [5]–[7], during which food is only available to animals at a particular period of time (such as during the day for nocturnal rodents). Under restricted feeding schedules, numerous physiological and metabolic functions, including locomotor activity, body temperatures, and insulin and corticosterone release, become entrained to the availability of food. Strong support for the existence of a food-entrainable timing system is provided by the circadian pattern of food anticipatory activity (FAA) rhythms that have been shown to develop in intact and SCN-ablated animals maintained on restricted feeding schedules [5], [6], [8]–[10].

Clock gene rhythms in the liver and cerebral cortex can also be entrained to restricted feeding stimuli within 2 days, i.e before FAA can develop, despite the locking of SCN activity to LD cues or SCN ablation [4], [11]–[13]. Dissociation of behavioral FAA rhythms and peripheral clock gene expression is not uncommon [14], [15]. Thus, the mechanisms by which feeding cycles can rapidly entrain peripheral oscillators and allow them to dissociate from the SCN remain unclear.

Glucose has been shown to induce phase shifts in the peripheral tissue clocks. In diabetic rats lacking insulin, the phase of cardiac circadian gene expression is advanced by approximately 3 h [16], suggesting that high blood glucose levels can cause phase shifts in the peripheral tissue clocks. Clock resetting by the down regulation of *Per1* and *Per2* has been observed in Rat-1 fibroblasts treated with glucose [17]. Glucose caused phase shifts in locomotor activity such as FAA if the rats were treated with a restricted feeding schedule [18]. The phase shift induced by glucose is probably not due to the higher energy content, since the consumption of vegetable oil did not result in a phase shift in rats on restricted feeding schedules [18]. Taken together, the data suggest that glucose may be at least one factor for peripheral tissue clock gene entrainment as well as FAA during restricted feeding schedules. Therefore, in the present experiment, we directly examined the effect of carbohydrates, including sugar and starch, on the entrainment of the circadian liver clock using *Per2*::luciferase knock-in mice.

Humans generally consume an appropriate balanced diet containing starch, protein, and oil, but not simple nutrients such as glucose. Therefore, to enable the application of our study results to humans, appropriate dietary guidelines were developed. In order to elucidate the role of nutrition in inducing phase shifts, the components of the AIN-93M diet were partially or completely substituted. Since glucose is a good candidate for inducing entrainment signals [18], we focused particularly on cornstarch and sugar. The dietary absorption rate of starch, i.e. the absorption rate of glucose into the bloodstream, is influenced by the ratio of amylose to amylopectin [19], [20]. Amylose-enriched starches are reported to increase blood glucose less than those enriched with amylopectin [21]. Thus, in the present experiment, we compared the effects of gelatinized cornstarch (GCS) and high-amylose cornstarch (HACS) on phase advance of the liver clock.

## Results

### Effect of food volume and duration of restricted feeding on the phase of the liver clock

**Figure 1:**
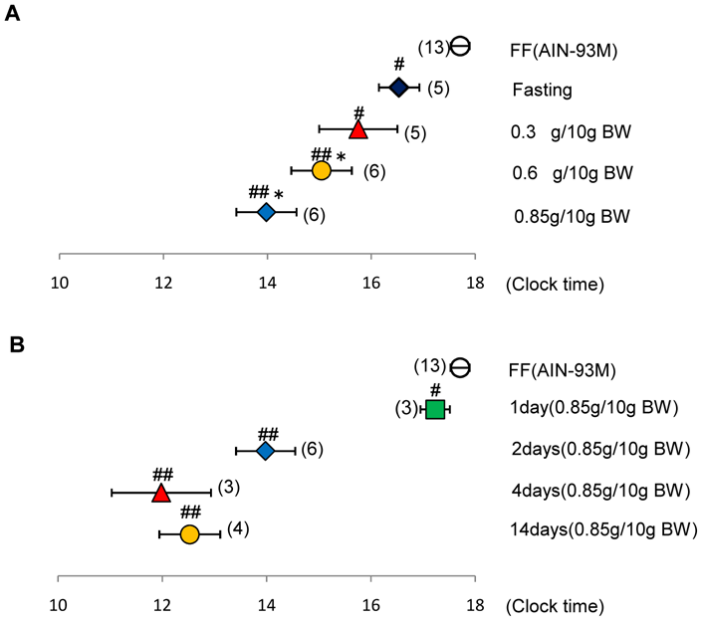
Effects of food volume and daily meal on the phase of the liver circadian clock. A: Mice were administered a diet tablet containing 0–0.85 g/10 g BW of the control AIN-93M diet for 2 days after 24-h food deprivation. Diet volume of 0.3 g/10 g BW for 2 days (0.3+0.3-g group); 0.6 g/10 g BW for 2 days (0.6+0.6-g group); or 0.6 g/10 g BW on the first day and 0.85 g/10 g BW on the second day (0.6+0.85-g group) was administered. B: Diets of 0.6 g/10 g BW on the first day (1-day group); 0.6 g/10 g BW on the first and 0.85 g/10 BW on the second day (2-day group); or 0.6 g/10 g BW on the first day followed by 0.85 g/10 g BW for the next 3 days (4-day group) was administered. Some mice were permitted free access to the control diet during ZT6–ZT10 (4 h) for 14 days (14-day group). The values indicate the mean±SEM. The horizontal axis indicates the projected zeitgeber time (pZT) at the peak of the bioluminescence rhythm. ZT0 is light-on time in the housing room prior to sacrifice of the mice. Fisher's PLSD test: #*P*<0.05, ##*P*<0.01 (vs. free feeding). *P<0.05 (vs. fasting). Two kinds of control experiments were prepared: Free feeding (open circle); fasting, 2-day fast (rhombus). The numbers in the parentheses indicate the number of tested mice.

Since restricted feeding for 4 h has been shown to cause phase advancement of the mouse liver clock [4], [11], [12],we measured the food intake for 4 h after a 24-h fasting. *Per2*:: luciferase mice (body weight, 23.8±0.8 g (range, 20–30 g); age, 10–18 weeks; N = 26) consumed the control diet: 1.4±0.11 g/mouse (0.60±0.040 g/10 g body weight [BW]) for 4 h on the first day and 2.0±0.13 g/mouse (0.85±0.047 g/10 g BW) on the second day after 24-h starvation. The quantity of food and eating speed did not differ between AIN-93M tablet food and the standard normal mouse diet (MF; Oriental Yeast Co. Ltd., Tokyo, Japan) (data not shown).Therefore, in the subsequent experiments, we administered an appropriately sized diet tablet to the mice after adjusting the food volume to the BW of each mouse. Zeitgeber time (ZT) 0 and ZT12 were the lights-on and lights-off times of the animal room, respectively. Under explant conditions, the term “projected ZT (pZT)” was used instead of ZT. Food volume of 0.3 g/10 g BW or 0.6 g/10 g BW for 2 consecutive days (0.6 g+0.6-g group) or 0.6 g/10 g BW on the first day and 0.85 g/10 g BW on the second day (0.6 g+0.85-g group) caused phase advancement of the liver clock in a food volume-dependent manner (F4,32 = 12.3, *P*<0.01, one-way ANOVA) (Fig. 1A). Fig. S1 shows an example of raw and detrended data of the bioluminescence rhythm of *Per2*::luciferase mice. Under our experimental conditions, fasting itself caused significant phase advancement of the liver clock (*P*<0.05 vs. FF, Fisher's PLSD test). Therefore, in the subsequent experiments, we evaluated the statistical differences by comparing the results with those of the fasting group. The liver clock phase advanced in a feeding day-dependent manner for the following diets: 0.6 g/10 g BW on the first day (1-day group); 0.6 g/10 g BW on the first and 0.85 g/10 BW on the second day (2-day group); or 0.6 g/10 g BW on the first day followed by 0.85 g/10 g BW for the next 3 days (4-day group) (Fig. 1B). According to a standard protocol for restricted feeding [12], AIM-93M tablets were freely given to mice during a period of 4 h (ZT6-ZT10) for 14 days (14-day group). The phase advances of these mice were similar to those of the 4-day group mice, suggesting that the 4-day feeding schedule in this study was sufficient to cause maximum phase advancement (Fig. 1B). The 2-day feeding schedule did not elicit a complete phase shift and therefore any potential diet-mediated enhancement of the shift could not be visualized. Thus, we selected the 2-day feeding schedule.

Under certain conditions the timing of the culture procedure itself may influence the phase of rhythms recorded in the tissue explants [22]. In the present experiments, culture time at ZT3 in free-feeding mice caused a phase peak at pZT 17.7±0.18 (N = 13) (approximately 15 h after explant), and culture time at ZT7 caused a phase peak at pZT 18.5±0.53 (N = 8) (approximately 11 h after explant). The culture time at ZT3 in the 2-day group caused a phase peak at pZT 14.0±0.56 (N = 6) (approximately 11 h after explants), and culture time at ZT23 in the 2-day group caused a phase peak at pZT 13.9±0.9 (N = 5) (approximately 15 h after explants). In both cases of free feeding and restricted feeding, the phase of the liver explants was independent of culture time. In the next experiment, we examined whether the phase advance by the 2-day feeding schedule stayed the same during food deprivation on the next day. The phase advance value of 2-day feeding of control food was pZT 14.6±0.8 (N = 4) and pZT 14.0±0.6 (N = 6) with or without next-day food deprivation, respectively. Values were almost identical between the two groups.

### Effect of complete substitution of each nutrient in the AIN-93M diet on the phase of the liver clock and relative blood glucose value

**Figure 2:**
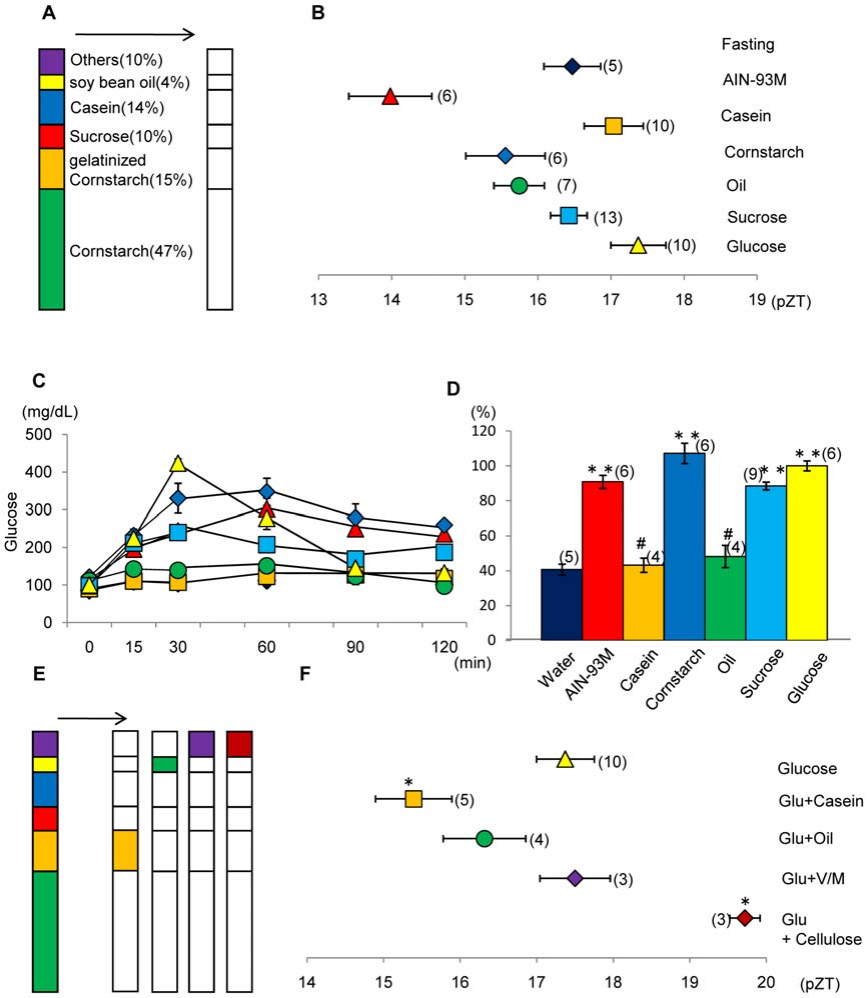
Effects of complete substitution by single nutrient components in the AIN-93M diet on the phase of the liver circadian clock and relative blood glucose value. A: Percent component of each nutrient in AIN-93M. Other contents were 3.5% AIN-93 mineral mixture, 1% AIN-93 vitamin mixture, 0.25% choline bitartrate, and 0.0008% tert-butyl hydroquinone. Substituted components are shown by the white column segments. B: Mice were administered a diet tablet containing 0.6 g/10 g BW of each nutrient on the first day and 0.85 g/10 g BW of each nutrient on the second day after 24-h food deprivation. Some mice were administered 0.3 mL of soybean oil on the first day and 0.43 mL of soybean oil on the second day after 24-h food deprivation. The values indicate the mean±SEM. The horizontal axis indicates the pZT at the peak of the bioluminescence rhythm. Fisher's PLSD test. *P<0.05 (vs. fasting); fasting, 2-day fast # *P*<0.05, ## *P*<0.01 (vs. AIN-93M). C: Time course of the blood glucose level after oral injection of 0.03 g/10 g BW of AIN-93M, 100% cornstarch, 100% casein, 100% sucrose, or 100% glucose or 0.015 mL/10 g BW of 100% soybean oil. For the control, 0.33 mL/10 g BW of water was administered. The vertical axis indicates the content of glucose (mg/dL). Symbol color of each treatment is same as column color in part D. D: Relative blood glucose value (% of blood glucose level produced by 100% glucose) of each nutrient group. This value was calculated by the following formula: 100× [AUC (area under the blood glucose concentration-time curve) during 2 h after each nutrient administration/AUC during 2 h after 100% glucose administration]. The values indicate the mean±SEM. Fisher's PLSD test. **P<0.01 (vs. water). # *P*<0.05 (vs. AIN-93M). The numbers in the parentheses indicate the number of tested mice. E: Percent component of each nutrient in AIN-93M. Substituted components are shown by the white column. F: Effect of combination of 100% glucose, 86% glucose +14% casein, 96% glucose +4% soybean oil, 95% glucose +5% cellulose, or 95.5% glucose +3.5% AIN-93 mineral mixture and 1% AIN-93 vitamin mixture on the liver circadian clock. Feeding schedule was the same as in Fig. 2B. The values indicate the mean±SEM. The horizontal axis indicates the pZT at the peak of the bioluminescence rhythm. Fisher's PLSD test. *P<0.05 (vs. 100% glucose). # *P*<0.05 (vs. AIN-93M). The numbers in parentheses indicate the number of tested mice. We used the same data of fasting and AIN-93M as mentioned in Fig. 1 to produce Fig. 2.

Fig. 2A shows the percent composition of each nutrient in the control AIN-93M diet. Each component was completely substituted as a single nutrient. The mice were administered diet tablets containing 0.6 g/10 g BW of each nutrient on the first day and 0.85 g/10 g BW of each nutrient on the second day after 24-h food deprivation. An insignificant, weak phase advance (*P*>0.05 vs. 2-day fasting group) of the liver clock was observed with 100% cornstarch (Fig. 2B). Similarly, an insignificant, weak phase advance of the liver clock was observed in mice (BW = 25 g) administered 0.3 mL of soybean oil on the first day and 0.43 mL of soybean oil on the second day as compared with mice from the 2-day fasting group (Fig. 2B). Diets containing 100% sucrose, 100% glucose, and 100% casein did not induce a phase advance of the liver clock (*P*>0.05, Fisher's PLSD test). The time course of blood glucose content measured at 15, 30, 60, 90, and 120 min after injection through an oral syringe of each nutrient is shown in Fig. 2C. The glucose content calculated by AUC (area under the blood glucose concentration-time curve) after administration of 100% glucose (0.033 g/10 g BW) was designated as 100% and relative values of glucose concentration (% of glucose) after injection of each nutrient is shown in Fig. 2D. The glucose concentration attained by administration of 100% cornstarch was slightly higher than that by glucose or AIN-93M. However, percent of glucose attained by 100% casein and 100% soybean oil was significantly lower than that attained by AIN-93M (P<0.01, Fisher's PLSD test). Administration of 100% sucrose induced high relative glucose values (Fig. 2C and D).

Since neither a 100% glucose diet nor a 100% sucrose diet could cause phase advances, other components contained in AIN-93M may cooperatively play a role in the action of sugar on phase advance of the liver clock (Fig. 2E). Combinations of 95.5% glucose with 4.5% vitamins/minerals mixture, 95% glucose with 5% cellulose, or 96% glucose with 4% soybean oil did not cause a phase advance (*P*>0.05 vs. 100% glucose) (Fig. 2F). However, the combination of 86% glucose with 14% casein significantly (*P*<0.05 vs. 100% glucose) increased the phase advance of the liver bioluminescence rhythm.

The free-running period of liver bioluminescence rhythm with the control diet AIN-93M (19.9±1.2, N = 5) was not significantly different from that of the free-feeding mice (19.0±0.8, N = 5). Therefore, we did not evaluate changes of the free-running period among different diet groups.

### Effect of partial substitution of cornstarch/sucrose in the AIN-93M diet on the phase of the liver clock and relative blood glucose value

**Figure 3:**
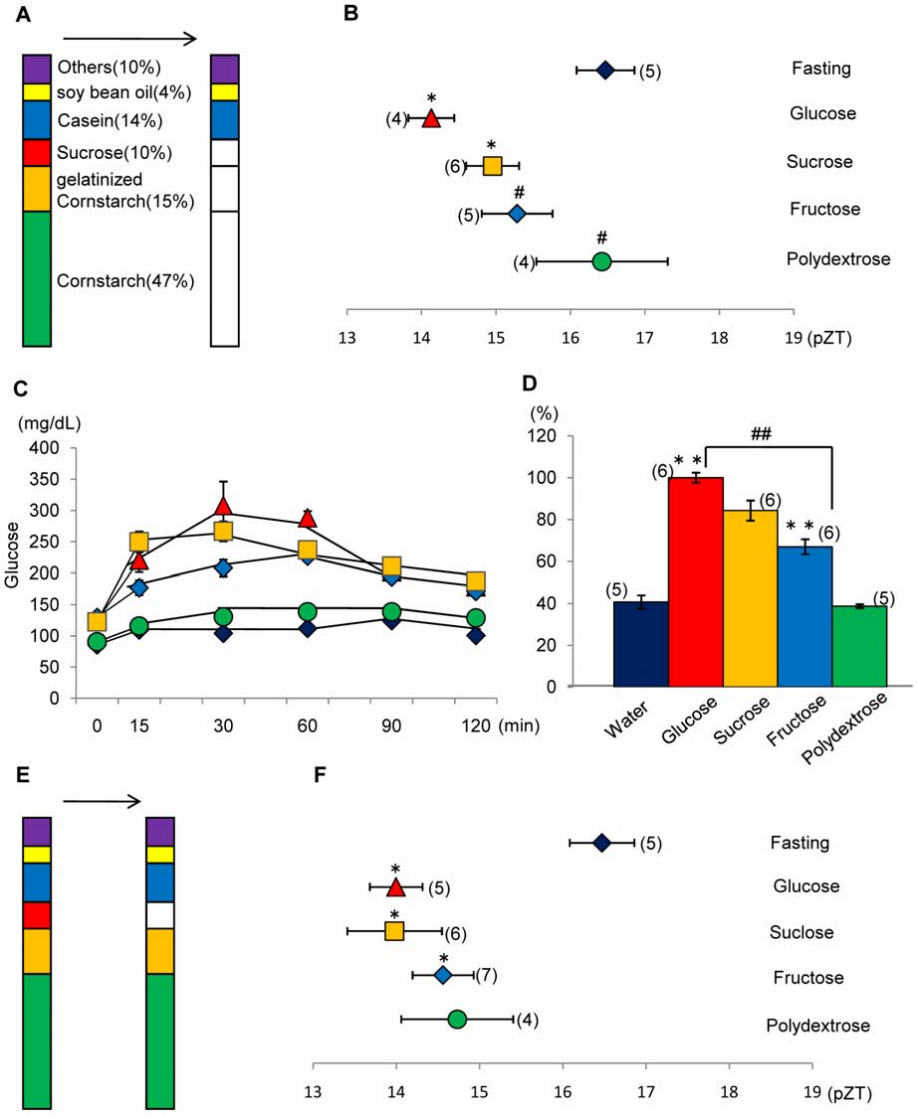
Effects of partial substitution by starch/sugar in the AIN-93M diet on the phase of the liver circadian clock and relative blood glucose value. A: Percent of each nutrient component in AIN-93M. The ‘other’ category comprised 3.5% AIN-93 mineral mixture, 1% AIN-93 vitamin mixture, 0.25% choline bitartrate, and 0.0008% tert-butyl hydroquinone. Substituted components are shown by the white column segments. B: Mice were administered a diet tablet containing 0.6 g/10 g BW of the substitute diet on the first day and 0.85 g/10 g BW of the substitute diet on the second day after 24-h food deprivation. Nutrient components of cornstarch, gelatinized cornstarch, and sucrose were replaced with glucose, sucrose, fructose, or polydextrose. The horizontal axis indicates the pZT at the peak of the bioluminescence rhythm. Fisher's PLSD test. *P<0.05 (vs. fasting). # *P*<0.05 (vs. glucose). C: Time course of the blood glucose level after oral injection of 0.03 g/10 g BW of each substituted nutrient. The vertical axis indicates the content of glucose (mg/dL). The values indicate the mean±SEM. Symbol color of each treatment is the same as column color in part D. D: Relative blood glucose value (% of glucose) of nutrient substituted group. Fisher's PLSD test. **P<0.01(vs. water). #*P*<0.05, ##*P*<0.01(vs. glucose). E: Percent component of each nutrient in AIN-93M. Substituted components are shown by white column. F: The component of 10% sucrose was substituted with 10% glucose, 10% fructose, or 10% polydextrose. The horizontal axis indicates the pZT at the peak of the bioluminescence rhythm. Fisher's PLSD test. *P<0.05 (vs. fasting). The numbers in parentheses indicate the number of tested mice. We used the same data for fasting and AIN-93M as mentioned in Fig. 1 to produce Fig. 3.

The combination of glucose with casein or oil was as good or better with respect to the phase-advancing effect; therefore, in the next experiment, we examined the effect of different sugars in AIN-93M on the phase of the liver clock. The nutrient components cornstarch, gelatinized cornstarch, and sucrose were replaced with glucose, sucrose, fructose, or polydextrose (Fig. 3A). Substitution with glucose, sucrose, fructose, or polydextrose caused increasing phase advances of the liver clock, respectively,(Fig. 3B). There were significant differences in the phase advance of the liver clock between the glucose/sucrose and fasting groups (*P*<0.05, Fisher's PLSD test); however, there were no significant differences in phase advance of the liver clock between the fructose/polydextrose and fasting groups. A time course of blood glucose levels and the percent of glucose value after oral injection of each of the substituted sugars are shown in Fig. 3C and D, respectively. The magnitude of phase shift and relative blood glucose value showed a positive correlation with the glucose substitution and resulted in a larger phase advance in the liver clock and a higher blood glucose value.

In order to confirm the effect of sugar on the phase shifts of the mouse liver clock, the 10% sucrose present in the control AIN-93M diet was substituted with 10% glucose, 10% fructose, or 10% polydextrose (Fig. 3E). When the percentage of cornstarch and gelatinized cornstarch in the AIN-93M diet was high (62%), the effect of substitution with glucose, fructose, or polydextrose was limited (Fig. 3F). Phase advancement of the liver clock was observed in order of increasing potency when glucose, sucrose, fructose, and polydextrose substitutions were made, respectively. However, there were no significant differences in the phase advancement of the liver clock among the substituted groups (F3,18 = 0.63, *P*>0.05, one-way ANOVA).

### Effect of cornstarch containing high amylose or low amylose on the phase of the liver clock

**Figure 4:**
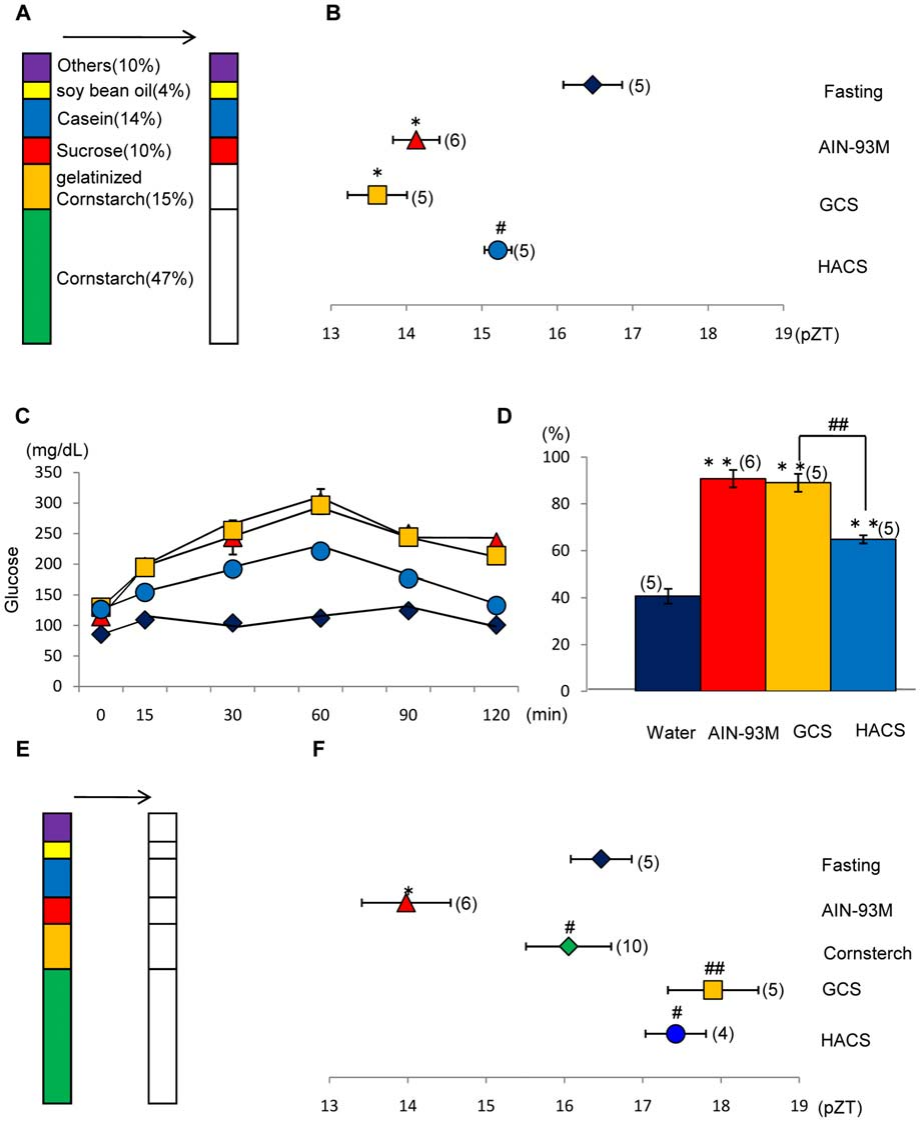
Effects of the substitution of cornstarch in AIN-93M by gelatinized cornstarch (GCS) or high-amylose cornstarch (HACS) on the phase of the liver circadian clock and relative blood glucose value. A: Percent component of each nutrient in AIN-93M. Substituted components are shown by the white column segments. B: Mice were administered a diet tablet containing 0.6 g/10 g BW of the substituted diet on the first day and 0.85 g/10 g BW of the substituted diet on the second day after 24-h food deprivation. The components of cornstarch and GCS in AIN-93M were replaced with GCS or HACS. The horizontal axis indicates the pZT at the peak of the bioluminescence rhythm. Fisher's PLSD test. *P<0.05 (vs. fasting). #*P*<0.05 (vs. GCS). C: Time course of the blood glucose level after oral injection of 0.03 g/10 g BW of each substituted nutrient. The vertical axis indicates the content of glucose (mg/dL). The values indicate the mean±SEM. Symbol color of each treatment is same as column color in part D. D: Relative blood glucose value (% of glucose) of the nutrient-substituted group. Fisher's PLSD test. **P<0.01 (vs. water), ##*P*<0.01 (vs. GCS). The numbers in parentheses indicate the number of tested mice. E: Percent component of each nutrient in AIN-93M. Substituted components are shown by white column segments. F: All nutrient components in AIN-93M were completely substituted with 100% cornstarch, 100% GCS, or 100% HACS. The horizontal axis indicates the pZT at the peak of the bioluminescence rhythm. Fisher's PLSD test. *P<0.05 (vs. fasting); fasting, 2-day fast # *P*<0.05, ## *P*<0.01 (vs. AIN-93M). The numbers in the parentheses indicate the number of tested mice. We used the same data of fasting or 100% cornstarch as mentioned in Fig. 2 to produce Fig. 4.

There are two different kinds of cornstarch: HACS and low-amylose cornstarch. In the present experiment, we substituted the components of cornstarch and gelatinized cornstarch in the AIN-93M diet with GCS or HACS (Fig. 4A). Substitution with GCS produced a stronger phase advance as compared to HACS with regard to the percent of glucose value (Fig. 4B, *P*<0.05, Fisher's PLSD test). As expected, the increase in blood glucose was faster and higher in the GCS group than in the HACS group (Fig. 4C and D). In order to confirm that 100% cornstarch caused a weak phase advance of the liver clock (Fig. 2B), we examined the effect of 100% GCS or 100% HACS on phase shift (Fig. 4E). Neither 100% GCS nor 100% HACS caused a phase advance of the liver clock (*P*>0.05 vs. fasting) (Fig. 4F). Thus, there were significant differences between the effects of AIN-93M containing cornstarch, GCS, or HACS (*P*<0.05, 0.01).

### Effect of AIN-93M, 100% glucose, or 86% glucose with 14% casein diet on feeding-induced FAA

**Figure 5:**
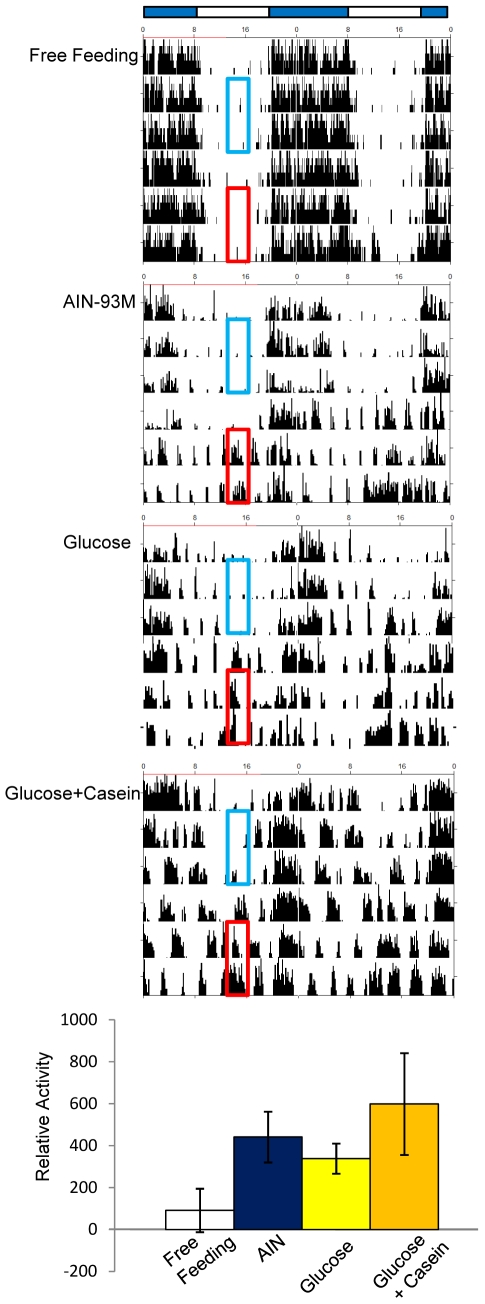
Restricted feeding-induced anticipatory activity (FAA) produced by AIN-93M, 100% glucose, or 86% glucose +14% casein diet. A: Examples of double-plotted actograms of free-feeding or restricted-feeding mice under LD cycle. Horizontal open and closed bars indicate the light and dark periods, respectively. Zeitgeber time (ZT) 0 indicates lights on and ZT12 lights off, under LD-cycle schedule. Blue area exhibits ZT3–ZT6 before restricted feeding for 2 days, and red area represents ZT3–ZT6 during restricted feeding for 2 days. Food was given at ZT6 for 2 days during red area after 24-h fasting. B: Percent change of anticipatory activity increase. Percent activity increase was calculated by the following formula: 100× (percent activity counts [ZT3–ZT6] of daily total activity during the 2-day restricted feeding schedule [red area]/those [ZT3–ZT6] during the 2-day free-feeding schedule [blue area]). The values indicate the mean±SEM. The numbers in parentheses indicate the number of tested mice. *P<0.05 from free-feeding schedule.

Although a diet of 100% glucose has been reported to cause FAA in the rat [18], our present results suggest that 100% glucose does not affect phase advance of the liver clock. Therefore, we evaluated whether 100% glucose caused FAA in mice. The same feeding schedule from the liver explants experiment was used for analysis of feeding-induced FAA. Restricted feeding of control diet AIN-93M for 2 days caused FAA in the mouse (Fig. 5), and similarly, a diet of 100% glucose or 86% glucose containing 14% casein also caused FAA (Fig. 5). Food anticipatory activity was evaluated by changes in activity counts in these groups compared with those occurring in the free-feeding schedule. Percent activity change in the free-feeding group was 91±100% (N = 9) but significantly higher in the restricted feeding groups of AIN-93M (441±121%, N = 4, *P*<0.05 vs. free-feeding), 100% glucose (338±72%, N = 4, *P*<0.05), and 86% glucose with 14% casein (598±242%, N = 3, *P*<0.05).

## Discussion

The present study suggests that a balanced diet, such as the control AIN-93M diet, is important for entrainment of the liver circadian clock. Surprisingly, with regard to starch/sugar, mouse diets with 100% glucose, 100% sucrose,100% cornstarch, 100% GCS, or 100% HACS do not cause significant phase advancement of the liver clock when compared with a 2-day fasting group. In addition to starch/sugar, 100% casein and 100% soybean oil also did not cause a significant effect on phase advance. Thus, the present study suggests that a balanced diet containing carbohydrate, protein, lipids, and vitamins/minerals may be effective for inducing phase shifts in the peripheral circadian clock. These data indicate that, simple diets such as 100% sugar, 100% protein, and 100% oil are inadequate for inducing entrainment signals. Therefore, we suggest that a balanced diet such as the control AIN-93M diet is good not only for maintaining the health and metabolism of mice but also for inducing entrainment signals in the peripheral circadian clock.

Although 100% glucose, 100% casein, 100% starch, and 100% soybean oil failed to cause a significant phase advance in the present study, 86% glucose with 14% casein caused a significant phase advance when compared to 100% sucrose alone. It has been observed that consumption of meals rich in protein and fat leads to secretion of gut hormones such as cholecystokinin (CCK), secretin, and peptide YY from the upper small intestine [23]–[25]. At present, we do not know the role of such gut hormones on phase shift of the liver clock; however, some studies have suggested a role of CCK in circadian systems [26], [27]. In one such behavioral experiment, it was shown that consumption of vegetable oil did not produce phase shifts in the activity rhythm of rats on a restricted feeding schedule [18]. Similarly, our present results suggest the inability of oil to cause a phase shift in the liver clock. The observation that 95% glucose with 5% soybean oil did not cause a phase advance of the liver clock could be attributed to potency differences of fat and oil or the level of protein and fat required for CCK release [23]–[25]. Two interesting findings strongly suggest that the digestive tract, particularly the jejunum, is an important site where the sensation of food is received as an entrainment signal for corticosterone rhythm. In one finding, the circadian corticosterone rhythm was entrained by restricted feeding in rats and maintained under enteral injection of artificial nutrition. However, it disappeared on middle cardiac vein injection of the same nutrition [28]. In a second finding, restricted feeding-entrained corticosterone rhythm was maintained under ileal resection in rats but disappeared under jejunal resection [29]. Our present results in the liver circadian clock support these studies since the AIN-93M diet, the partial substitution diet, and the glucose with casein diet caused significant and large phase advances of the liver clock rhythm. These results suggest that at minimum a combination of starch/sugar with protein may be necessary to produce adequate phase shift of the liver clock. Conversely, vitamins/minerals or cellulose contained in AIN-93M may not contribute to an AIN-93M-induced phase advance since the combination of glucose with vitamins/minerals or glucose with cellulose did not cause a phase advance.

In the current study we have found that starch/sugar in the AIN-93M diet are important mediators of liver phase advancement. An AIN-93M diet containing a substitution of polydextrose for starch/sugar resulted in a failure of liver phase advancement. The inability of the polydextrose diet to produce a phase shift could have been due to its relatively low caloric load. Partial substitution of glucose, sucrose, fructose, and polydextrose resulted in a positive effect on phase advancement with respect to the order of relative blood glucose values. In addition, two types of cornstarch were used to further support these findings [30]. Substitution of cornstarch by HACS caused a significantly weaker phase advance when compared to that in the control cornstarch and GCS-substituted groups. In fact, the blood glucose value was significantly higher in the GCS group as compared to the HACS group. These results suggest that a diet containing starch/sugar that produces high blood glucose values may be beneficial for phase shift of the liver clock if fed to animals in the context of a balanced diet. The data also underscore the important effect that an increase in blood glucose concentration has on the phase advance of the liver clock.

Rat-1 fibroblasts treated with glucose have been shown to exhibit clock resetting by the down-regulation of *Per1* and *Per2* [17]. However, our data show that 100% glucose does not cause phase advance. The difference between our present results and that observed by Hirota et al. may be attributable to the experimental design, which was *in vitro* and *in vivo*, respectively. Recently, it has been reported that fasting up-regulated *Per1* and down-regulated *Per2*, *Dec1*, and *Bmal1* in the mouse liver and that re-feeding prevented the fasting-induced change of gene expression [31], [32]. Thus, up and down changes of gene expression by a balanced diet (normal mouse chow) may not be reproduced by a simple diet of 100% glucose. Future experiments will compare the clock gene-expression pattern in the liver between a balanced diet and a 100% sucrose diet. In one behavioral study, glucose shifted the phase of FAA when the rats were subjected to a restricted feeding schedule [18]. We have found that in addition to the AIN-93M diet, 100% glucose and 86% glucose with 14% casein caused FAA under a 2-day restricted feeding schedule, suggesting that 100% glucose has the ability to produce FAA without affecting the liver clock. Dissociation of behavioral FAA rhythms and peripheral clock gene expression has been reported [14], [15]. Although the mechanism of such dissociation by glucose is not elucidated in the present study, this is the first successful report of dissociation by diet.

Given the present results, it is not clear whether the effects we observed on the liver clock were specific effects on a food-entrainable circadian pacemaker or were a more global effect on the circadian system. If restricted feeding with different diets had global effects, then we may see shifts in the SCN. However, if the effects were specific to the food-entrainable oscillator, then we would not expect to see shifts in the SCN. Although previous papers have suggested that restricted feeding does not cause a phase shift of the SCN clock [4], [11], [12], our future studies will examine whether balanced food, substituted food, or simple nutrients can cause a shift of the SCN clock.

Previous studies have shown that the volume of food can produce large phase shifts in the FAA rhythm in rats in a food volume-dependent manner, while substitution of the diet with non-nutritive bulk failed to produce phase shifts [33]. When rats were entrained to 20 g food/day, approximately 6 g was necessary to produce phase shifts [33]. In our present study, we found that phase advance of the liver clock was dependent on the volume of food (0.7, 1.4, or 2.0 g/day). Mice consume 4–6 g food/day under free-feeding conditions and 1.4 g food/day is required to cause phase shifts. Thus, we estimate that at least 30% of the food intake under free-feeding is required to cause phase shifts in the liver clock in both mice and rats. After feeding the mice 1.4 g food/day, our data show a significant phase advance of the bioluminescence rhythm on the second day but not the first. A previous study has also reported clear phase shifts induced by restricted feeding schedules on the second day of daytime feeding [34]. Thus, entrainable stimulation for at least 2 days may be necessary to produce a phase advance and entrainable stimulation for 4 days is needed to produce a maximum phase advance of the liver clock.

In summary, the present results strongly suggest that a balanced diet is important for restricted feeding-induced entrainment of the peripheral clock rhythm and that balanced foods that lead to high blood glucose levels are suitable for peripheral clock entrainment. In contrast, simple foods such as 100% sugar, 100% protein, and 100% oil are not suitable for the generation of entrainment signals. These findings may aid in dietary recommendations for on-board meals served to air travelers and shift workers to reduce jet lag-like symptoms.

## Materials and Methods

### General condition of the animals and housing

*Per2::*luciferase mice (courtesy, Dr. Joseph Takahashi, Northwestern University, Evanston, IL, USA) [35] were bred in-house with C57/B6 (Clea Japan, Tokyo). Zeitgeber time (ZT) 0 and ZT12 were the lights-on and lights-off times, respectively. Under explant conditions, we used the term “projected ZT (pZT)” rather than ZT. The light intensity at the surface of the cages was approximately 100 lux. In the present experiments, we used *Per2::*luciferase homozygous and heterozygous male mice. We did not observe any differences in the peak time of bioluminescence in the liver between the homozygous and heterozygous mice (homozygous: 17.9±0.32 pZT, N = 7; heterozygous: 18.1±0.26 pZT, N = 8). The environmental conditions in the animal room were controlled at a temperature of 22±2°C, humidity of 60±5%, and a 12-h light/12-h dark cycle (lights on from 08:00 to 20:00 h) Before the restricted feeding experiment, the mice were fed a normal commercial diet (Catalog # MF; Oriental Yeast Co. Ltd., Tokyo, Japan), and water was available *ad libitum*. Experimental animal care was conducted under permission from the Committee for Animal Experimentation of the School of Science and Engineering at Waseda University (permission # 09A11).

### Preparation of food tablets for restricted feeding

For the restricted feeding experiments, we prepared diet tablets of 0.5–2 g depending on the mouse BW using a tableting machine (HANDTAB-100; Ichihashi-seiki, Kyoto, Japan). For the control diet, AIN-93M formula diet (Oriental Yeast Co. Ltd., Tokyo, Japan; composition: 14% casein, 0.3% l-cysteine, 47% cornstarch, 15% gelatinized cornstarch, 10% sucrose, 4% soybean oil, 5% cellulose powder, 3.5% AIN-93 mineral mixture, 1% AIN-93 vitamin mixture, 0.25% choline bitartrate, and 0.0008% tert-butyl hydroquinone) was prepared. In each figure we show the nutritive content of the control and substitution diets. For the substitution experiments, each component of the diet was substituted by another nutrient. In some experiments, 100% casein, 100% cornstarch, 100% sucrose, 100% glucose, and 100% soybean oil were used. In order to allow for the mice to adapt to the control diet, their diet was changed from normal to the control AIN-93M diet 3–4 days prior to the experiment. After 24-h food deprivation from the control diet, each mouse was provided a food tablet at ZT6 using an automatic food vending machine. Four hours after providing a food tablet, food consumption was checked. Some mice ate the entire tablet within 4 h, but some mice did not, depending on palatability. If the remaining food pellet comprised more than 0.09 g/10 g BW (2 SEM of the average food intake of normal food; see Results section) on day 2, the mice were not used for the *in vitro* experiments.

Pre-gelatinized cornstarch (GCS; Nisshoku Alstar E) and high-amylose cornstarch (HACS; Nisshoku High-Amylose Starch) were purchased from Nihon Shokuhin Kako (Tokyo, Japan). The characteristics of these cornstarches have been published previously [30]. Their amylose contents were 26 and 68 g/100 g, respectively. The amount of resistant starch in GCS and HACS was 5.6 and 50.7 g/100 g, respectively, and the dietary fiber content of GCS and HACS was 0.1 and 19.3 g/100 g, respectively [30]. Thus, in the current study we compared the influence of GCS and HACS on phase advance of the liver clock.

### Preparation and measurement of bioluminescence from *Per2*::luciferase mice

Following the feeding experiment, *Per2:*:luciferase mice were sacrificed at ZT3 for recording of the bioluminescence rhythmicity in the liver. A block of the liver was rapidly removed from each of the mice and placed in ice-cold Hanks' balanced salt solution (pH 7.2; Sigma-Aldrich, St. Louis, MO, USA). The liver tissues were cut into pieces with small scissors and explanted in a 35-mm Petri dish, sealed with parafilm (Sigma-Aldrich), and cultured with 1.3 ml DMEM (Invitrogen, Carlsbad, CA) supplemented with NaHCO_3_ (2.7 mM), HEPES (10 mM), kanamycin (20 mg/L; Sigma-Aldrich), insulin (5 µg/mL; Sigma-Aldrich), putrescine (100 µM; Sigma-Aldrich), human transferrin (100 µg/mL; Sigma-Aldrich), progesterone (20 nM; Sigma-Aldrich), sodium selenite (30 nM; Sigma-Aldrich), and 0.1 mM d-luciferin Na salt (Invitrogen) The concentration of glucose (4.5 g/L) is much higher than serum glucose (see Results section), therefore it is important to note that all *in vitro* experiments were conducted under such conditions. The cultures were incubated at 37°C and bioluminescence was monitored at 10-min intervals for 1 min using a dish-type luminometer (LumiCycle; Actimetrics, Wilmette, IL, USA).

### Assessment of the circadian periods and phases of the liver clock

First, the original data (1-min bins) were smoothed by an adjusting-averaging method with 2-h running means, as previously described [36]. Second, the data set was detrended by subtracting the 24-h running average from the raw data using R software [37], [38] (R development Core Team; http://www.r-project.org/). The program was developed by Mr. Tuyoshi Yaita and Dr. Shigenobu Shibata (Waseda University, Tokyo, Japan). The peaks were defined as points at which the bioluminescence was higher than that on both sides of the points and were confirmed from the waveform (supporting Fig. S1). Typically, the second peak is considered for the determination of the peak phase time because the first peak is sometimes affected by the movements of the culture dish. The period of *Per2*::luciferase activity (recorded from 24 to 72 h *in vitro*) was assessed for each liver culture sample and calculated by averaging the time periods between the first and second peaks and between the second and third peaks.

### Time-course measurement of blood glucose concentration and relative blood glucose values

Each tablet was dissolved in water at a concentration of 0.1 g/mL except for the soybean oil liquid. For this experiment, we administered 0.33 mL/10 g BW through an oral syringe since the onset of food intake needed to be adjusted for a time-course measurement of blood glucose levels. In consideration of the suspension ability or aqueous solubility of the diet, the concentration was set as 0.1 g/mL solution. Each of the diet solutions was injected at a dose of 0.033 g/10 g BW and this value was approximately 1/10 of the tablet diet (refer to Results). In human experiments, the glucose relative value (%) is calculated using the following formula: 100× (blood glucose up to 2 h after food intake/total blood glucose up to 2 h after intake of the same amount of glucose) [19]. Therefore, in the present mouse experiment, blood glucose was measured at 15, 30, 60, 90, and 120 min after oral intake. The AUC (area under the blood glucose concentration-time curve) was calculated for each diet including 100% glucose, and then relative glucose values (%) for each diet were calculated by 100× (mean AUC for each diet/mean AUC for 100% glucose). Fig. 2B shows the time course of glucose content in the tail blood after bolus injection of 100% glucose or water to fasting mice. Thus, mean AUC for 100% glucose was designated as 100% (Fig. 2C). For the blood glucose measurement, the tail vein of each mouse was cut and the glucose content was measured by using a Glucose PILOT kit (Aventir Biotech, LLC, Carlsbad, CA, USA) under light ether anesthesia. The range of blood glucose that could be measured using this kit was between 20 mg/dL and 600 mg/dL.

### Locomotor activity analysis

For assessment of FAA, mice were housed individually and general locomotor activity was recorded with an infrared radiation sensor (F5B; Omron, Tokyo, Japan) and analyzed with CLOCKLAB software (Actimetrics, Wilmette, IL, USA). Details of the protocol for measurement of activity have been published previously [39]. The daily counts were calculated as the number of the sensor counts per 6 min. Percent activity increase (anticipation) was calculated by the following formula: 100× (percent activity counts [ZT3–ZT6] of daily total activity during the 2-day restricted feeding schedule/those [ZT3–ZT6] during the 2-day free feeding schedule).

### Statistical Analysis

The values are expressed as the mean±standard error of the mean. For statistical analysis, one- or two-way analysis of variance (ANOVA) was applied and post hoc analysis was conducted with Fisher's PLSD test.

## Supporting Information

Figure S1 Representative expression rhythms of the liver Per2::luciferase bioluminescence in mice under the restricted feeding schedule. A: Raw experimental data of the mice liver under free feeding, 2-day fasting, or re-feeding conditions. For the re-feeding schedule, the mice were administered 0.6 g/10 g BW of food on the first day, and 0.85 g/10 g BW of food on the second day at ZT6 after 24-h fasting. B: The detrended data. The detailed method of detrending has been described in the text.
(9.41 MB TIF)
