# Supplementary figures and images for: A Balanced Diet Is Necessary for Proper Entrainment Signals of the Mouse Liver Clock
Source: PLoS One. 2009 Sep 7;4(9):e6909. doi: 10.1371/journal.pone.0006909 (PMC2734168; doi:10.1371/journal.pone.0006909)

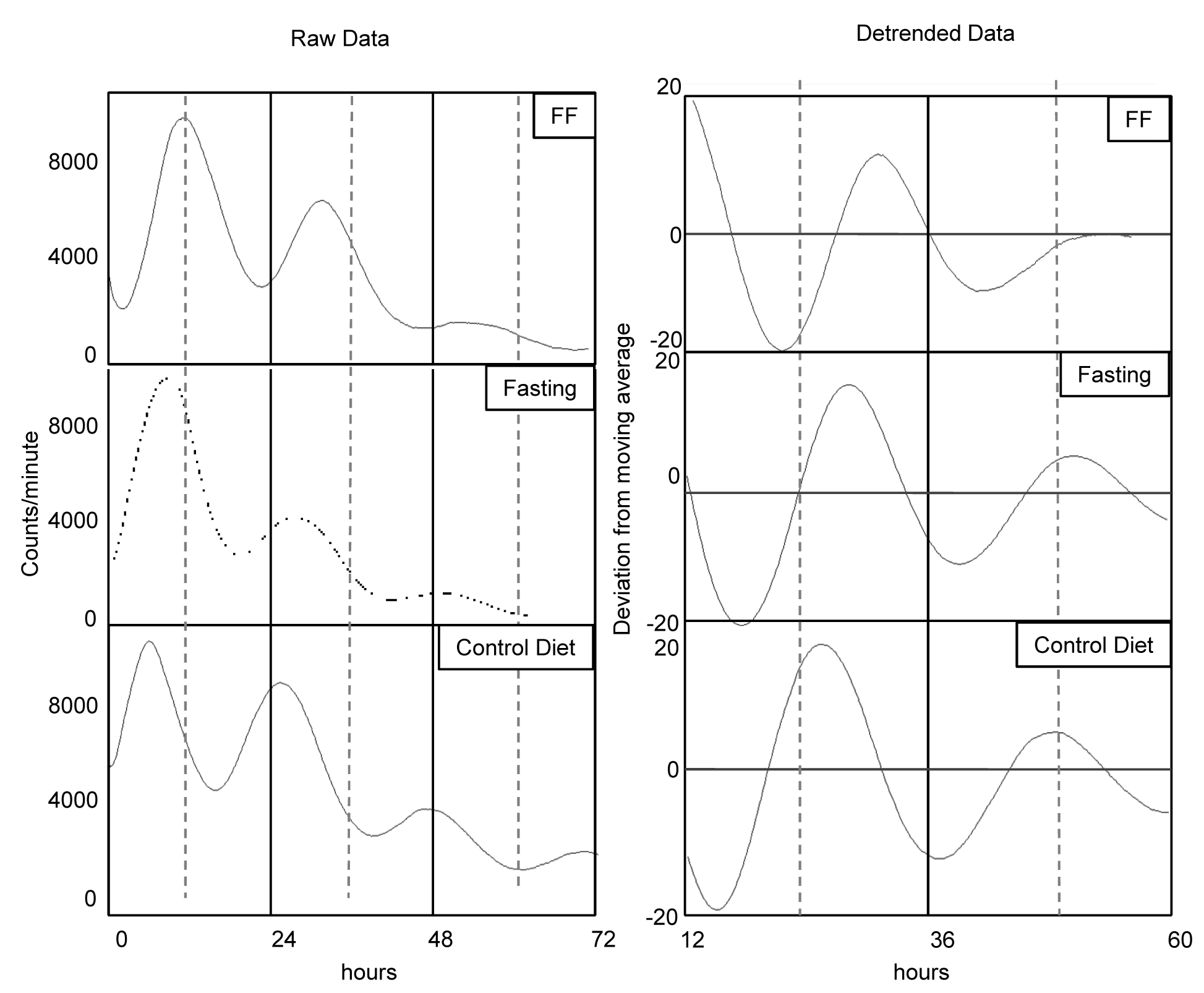

Supplement: Figure S1 — Representative expression rhythms of the liver Per2::luciferase bioluminescence in mice under the restricted feeding schedule. A: Raw experimental data of the mice liver under free feeding, 2-day fasting, or re-feeding conditions. For the re-feeding schedule, the mice were administered 0.6 g/10 g BW of food on the first day, and 0.85 g/10 g BW of food on the second day at ZT6 after 24-h fasting. B: The detrended data. The detailed method of detrending has been described in the text. (9.41 MB TIF) [file pone.0006909.s001.tif]
